# Differential methylation at *MHC* in CD4^+^ T cells is associated with multiple sclerosis independently of *HLA-DRB1*

**DOI:** 10.1186/s13148-017-0371-1

**Published:** 2017-07-18

**Authors:** Vicki E. Maltby, Rodney A. Lea, Katherine A. Sanders, Nicole White, Miles C. Benton, Rodney J. Scott, Jeannette Lechner-Scott

**Affiliations:** 1 Centre for Information Based Medicine, Hunter Medical Research Institute, Locked Bag 1, Hunter Region Mail Centre, Newcastle, 2310 Australia; 2 School of Biomedical Sciences and Pharmacy, University of Newcastle, Newcastle, Australia; 3 Institute of Health and Biomedical Innovation, Queensland University of Technology, Brisbane, Australia; 4 Faculty of Health Sciences and Medicine, Bond University, Robina, Australia; 5 Division of Molecular Genetics, Hunter Area Pathology Service, Newcastle, Australia; 6 Department of Neurology, Division of Medicine, John Hunter Hospital, Newcastle, Australia

## Abstract

**Background:**

Although many genetic variants have been associated with multiple sclerosis (MS) risk, they do not explain all the disease risk and there remains uncertainty as to how these variants contribute to disease. DNA methylation is an epigenetic mechanism that can influence gene expression and has the potential to mediate the effects of environmental factors on MS. In a previous study, we found a differentially methylation region (DMR) at MHC *HLA-DRB1* that was associated within relapsing-remitting MS (RRMS) patients in CD4^+^ T cells. This study aimed to confirm this earlier finding in an independent RRMS cohort of treatment-naïve female patients.

**Methods:**

Total genomic DNA was extracted from CD4^+^ T cells of 28 female RRMS and 22 age-matched healthy controls subjects. DNA was bisulfite-converted and hybridised to Illumina 450K arrays. Beta values for all CpGs were analysed using the DMPFinder function in the MINFI program, and a follow-up prioritisation process was applied to identify the most robust MS-associated DMRs.

**Results:**

This study confirmed our previous findings of a hypomethylated DMR at *HLA-DRB1* and a hypermethylated DMR at *HLA-DRB5* in this RRMS patient cohort. In addition, we identified a large independent DMR at MHC, whereby 11 CpGs in *RNF39* were hypermethylated in MS cases compared to controls (max. ∆beta = 0.19, *P* = 2.1 × 10^−4^). We did not find evidence that SNP genotype was influencing the DMR in this cohort. A smaller *MHC* DMR was also identified at *HCG4B*, and two non-MHC DMRs at *PM20D1* on chr1 and *ERICH1* on chr8 were also identified.

**Conclusions:**

The findings from this study confirm our previous results of a DMR at *HLA-DRB1* and also suggest hypermethylation in an independent MHC locus, *RNF39*, is associated with MS*.* Taken together, our results highlight the importance of epigenetic factors at the MHC locus in MS independent of treatment, age and sex. Prospective studies are now required to discern whether methylation at MHC is involved in influencing risk of disease onset or whether the disease itself has altered the methylation profile.

**Electronic supplementary material:**

The online version of this article (doi:10.1186/s13148-017-0371-1) contains supplementary material, which is available to authorized users.

## Background

Multiple sclerosis (MS) is an autoimmune disease characterised by lymphocyte-mediated inflammation causing demyelination and axonal degeneration. The underlying pathogenesis of MS remains unclear, but the risk of developing MS is influenced by a combination of genetic predisposition and environmental exposures. Several large genome-wide association studies (GWAS) have clearly identified major histocompatibility complex (MHC) region on chromosome 6p21, as the most important effect size, with the majority of the single-nucleotide polymorphisms (SNPs) that reached statistical significance falling within this region [1, 2]. The largest effect single-nucleotide polymorphism is the well-established human leukocyte antigen (HLA) class II region (HLA-DRB1*1501 in particular). Despite the significant contribution of the MHC region to MS risk and the large-scale GWAS study, there remains a large proportion of unexplained heritability in terms of MS risk [1, 3].

The main environmental exposures presumed to modify MS risk are smoking, sunlight exposure and Epstein-Barr virus (EBV) (reviewed in [4]). Epigenetics can influence the genome without changes to the DNA sequence. Environmental exposures such as smoking and sunlight exposure have been shown to be mediated by epigenetic mechanisms, providing a plausible link between environmental factors and disease [5, 6]. One such epigenetic mechanism is DNA methylation, which is the addition of a methyl group to CpG dinucleotides. We, and others, have used genome-wide DNA methylation technologies to assess differentially methylated regions (DMRs) of CD4^+^ and CD8^+^ T cells in relapsing-remitting multiple sclerosis (RRMS) patients compared to healthy controls [7–10]. These studies have found inconsistent and/or conflicting results [7, 8, 10]. Both groups found significant differences between CD4^+^ and CD8^+^ T cells [7, 8, 10]; however, our studies found a striking methylation signal located on chromosome 6p21 with a peak signal at *HLA-DRB1*, present in relapsing-remitting patients compared to healthy controls [7]. We found this signal to be specific to CD4^+^ T cells, but Bos and colleagues did not see this in either CD4^+^ or CD8^+^ T cells [8, 10].

The differences between the studies could be explained in several ways. In our previous study, the majority of patients were on some type of immunomodulatory treatment as opposed to the Bos et al. study, who used  treatment naïve patients. Also, there were differences in analysis methods in regard to filtering of probes contained within the SNP heavy region in which the methylation signal is contained in. In an effort to determine if we could replicate our initial results under a more tightly controlled study design, we performed a genome-wide DNA methylation study of CD4^+^ T cells in a new cohort of RRMS patients and healthy controls. This cohort has a more accurately age- and gender-matched control cohort and is comprised entirely of treatment-naïve patients or patients who have been free from immunomodulatory therapy for at least 3 months. Identifying epigenetic loci associated with MS, independent of treatment or SNP effects, could reveal potentially modifiable targets for environmental exposures or new drug design as well as identify potential markers for blood-based biomarkers of MS risk and treatment response.

## Methods

### Subject recruitment

Whole blood was initially collected from 28 female RRMS patients and 28 age-matched female healthy donors (Table 1). We chose to focus only on females to reduce potential sex effects and because female patients are more at risk of RRMS compared to males. All patients were diagnosed with MS according to the McDonald criteria [11] and were treatment naïve (19 patients) or had not taken immunomodulatory or steroid treatment for a minimum of 3 months (9 patients). The purpose of this design feature was to control for treatment effects as much as possible. Healthy control samples were collected from volunteers off the Hunter Medical Institute Research Register.

**Table 1:** Clinical characteristics of the cohort

	HC	RRMS
Total	22	28
Age in years (mean ± SD)	38.2 ± 11.1	38.6 ± 12.7
EDSS (mean ± SD)	n/a	2.1 ± 1.5
Disease duration in years (mean ± SD)	n/a	7.6 ± 11.4

*HC* health control, *RRMS* relapsing-remitting multiple sclerosis, *EDSS* Expanded Disability Status Scale

### Blood sample processing and DNA methylation arrays

Peripheral blood mononucleocytes (PBMCs) were isolated from whole blood by density gradient using Lymphoprep (Stemcell Technologies, Canada) following standard laboratory procedures. Total CD4^+^ T cells were extracted from the PBMC population using EasySep negative magnetic separation according to the manufacturers’ instructions (Stemcell Technologies, Canada). After isolation, cell purity was assessed by flow cytometry. Cells were stained with a FITC-conjugated CD4 antibody (60016F1 StemCell Technologies, Canada) and collected on a BD FACSCanto II flow cytometer, then analysed using FACSDiva software (BD Biosciences). All samples met the minimum purity cutoff of 90%. DNA was extracted with the Qiagen microDNA extraction kit (QIAGEN, USA) and biosulphite converted using the MethylEasy Xceed kit according to the manufacturers’ instructions. Converted DNA was then applied to the Illumina Infinium Human450K Beadchip methylation arrays (service provided by Diamantina).

### Data analysis

An in-house data analysis pipeline that used a combination of R/Bioconductor and custom scripts was designed. Illumina 450k raw intensity data (idat files) were parsed into the Bioconductor MINFI package [12]. Methylation data was background-corrected and quantile-normalised according to MINFI routines. Data was cleaned by removing (failed) CpG probes for which the intensity of both the methylated and unmethylated probes was <1000 units across all samples. A threshold of 1000 units was selected based on the profile of the available negative control probes. Y chromosome probes were filtered out. All probe sequences were mapped to the human genome (buildHg19) using BOWTIE [13] to identify potential hybridisation anomalies. In total, 33,457 CpG probes were identified to align to the human genome multiple times and were filtered out of subsequent analysis. We chose to retain probes containing SNPs and filter these out post hoc where appropriate.

Measures of methylation level (*β* values) were produced for each CpG probe and ranged from 0 [completely unmethylated] to 1 [completely methylated]. To identify differentially methylated positions (DMPs) associated with MS subtypes in this cohort, the DMPFinder function was implemented. This calculates an *F* statistic for each CpG by comparing means between the case and control group. Subtracting control mean from case mean produces a Δbeta score—a measure of differential methylation ranging from −1 to 1. The Δbeta score can be broadly interpreted as percentage up or down methylation in cases compared to controls (or effect size).

Given the relatively modest sample size, this study was underpowered to detect significant DMPs at the methylome-wide level and thus we used a series of prioritisation steps to identify the most robust loci. Specifically, a DMP was defined as containing CpGs (i) that yielded a *P* < 0.05, i.e. nominally associated with MS, and (ii) that yielded a ∆beta of ±0.1, i.e. a relatively large differential methylation. Subsequently, a differentially methylated region (DMR) was defined as a DMP (iii) that had ≥2 adjacent CpGs within 1000-bp physical distance and (iv) whereby adjacent CpGs yielded a ∆beta in the same direction, i.e. all three CpGs in the DMR were consistently hypo- or hyper-methylated.

Gene set (or pathways) analysis was using the over-representation analysis (ORA) routing of the WebGestalt server (www.webgestalt.org/). Specifically, we entered our gene list into the gene ontology—biological processes database query—and used the default ORA parameters to explore whether our gene lists tended to favour a particular pathway(s) at a false discovery rate (FDR) of 5%.

## Results

### Identification of DMPs and DMRs associated with MS

We analysed CpG methylation data specifically for CD4^+^ T cells obtained from 28 female RRMS patients—all treatment naïve—and 22 healthy age- and sex-matched control subjects. NB: Six control subjects were dropped due to poor quality methylation data. Clinical characteristics of the patient group are shown in Table 1.

Following the QC steps, methylation data for 445,787 CpGs were analysed using DMPFinder to identify DMPs associated with MS. A CpG prioritisation process was used to select the most robustly associated DMPs. First, all CpGs yielding a *P* < 0.05 were selected as being nominally associated with MS. Secondly, of the resultant list, only CpGs yielding a ∆beta of ±0.1 were selected. These steps resulted in 275 DMPs localising to 139 different genes and 136 unannotated genomic locations (see Additional file 1). Of the 275 DMPs, 134 (49%) were hypermethylated and 141 (51%) were hypomethylated. The top hit was for cg10568066 in the ring finger protein 39 (*RNF39*) gene whereby the CpG was hypermethylated in the case group compared to controls (∆beta = 0.19, *P* = 2.1 × 10^−4^). A gene ontology (pathways) analysis of the 134-gene set revealed that the biological process involving “regulation of GTPase activity” was over-represented by the gene list—GO:0043087, FDR = 0.015 (Additional file 2). GTPases are involved in signal transduction and cell differentiation and have been the target of many studies of MS.

Of the 275 DMPs, 14% are located within the MHC region. Importantly, nine of these were identified in our previous study and five are located in the main DMR we previously reported to be associated with MS in CD4^+^ T cells, i.e. *HLA-DRB1* [7]. In addition to the previously identified sites, we also identified three new CpGs within the *HLA-DRB1* DMR. All except one are located within the same 400-bp region. We also observed hypermethylated DMPs at *HLA-DRB5* and *HLA-DQB1* consistent with our previous study (Additional file 1) [7]. At the *HLA-DRB5* region, we identified one previously identified probe plus an additional two sites. We did not see any change in methylation at the CpG sites in *HLA-DRB6* previously identified, but did find two new hypermethylated sites (Additional file 1).

To identify DMRs, we focused only on those DMPs (i) that had ≥2 adjacent CpGs located within 1000-bp physical distance and (ii) whereby adjacent CpGs yielded a ∆meth in a consistent direction, i.e. all CpGs in the DMR were either hypo- or hyper-methylated. Table 2 shows the 33 DMPs representing six DMRs identified in this study. The largest DMRs were located within the MHC region on Chr 6. In addition to the DMR at *HLA-DRB1*, there was another large DMR in MHC region at *RNF39*. For this DMR, we observed 11 CpGs that were hypermethylated in the MS case group compared to the control (average ∆beta = −0.13). These CpGs were tightly clustered within a 346-bp region of the gene body. This region spans the boundary between intron 3 and exon 4 and spreads into exon 4 of *RNF39*.

**Table 2:** Thirty-three DMPs representing six DMRs associated with MS

CpG ID	Chr	Pos (bp)	DMR	Gene	Region	F stat	*P* value	Control	Case	∆beta
cg17178900	1	205,818,956	1	*PM20D1*	Body	6.54	0.014	0.470	0.646	0.176
cg26354017	1	205,819,088	1	PM20D1	1stExon	6.50	0.014	0.411	0.599	0.188
cg14159672	1	205,819,179	1	*PM20D1*	1stExon	6.09	0.017	0.444	0.616	0.173
cg14893161	1	205,819,251	1	*PM20D1*	5′UTR	7.42	0.009	0.328	0.498	0.169
cg11965913	1	205,819,406	1	*PM20D1*	TSS200	5.38	0.025	0.303	0.467	0.164
cg24503407	1	205,819,492	1	*PM20D1*	TSS1500	6.38	0.015	0.404	0.560	0.157
cg25644740	6	29,894,152	2	*HCG4P6*	TSS1500	5.93	0.019	0.448	0.612	0.164
cg04520169	6	29,894,195	2	*HCG4P6*	TSS1500	7.27	0.010	0.377	0.524	0.148
cg23237314	6	29,894,197	2	*HCG4P6*	TSS1500	6.97	0.011	0.374	0.492	0.117
cg08491487	6	30,039,130	3	*RNF39*	Body	10.02	0.003	0.078	0.181	0.103
cg00947782	6	30,039,142	3	RNF39	Body	12.34	0.001	0.102	0.210	0.108
cg03343571	6	30,039,175	3	*RNF39*	Body	13.64	0.001	0.212	0.334	0.122
cg13185413	6	30,039,202	3	*RNF39*	Body	11.78	0.001	0.206	0.326	0.119
cg09279736	6	30,039,403	3	*RNF39*	Body	14.19	0.000	0.287	0.404	0.117
cg07382347	6	30,039,408	3	*RNF39*	Body	13.22	0.001	0.153	0.295	0.142
cg13401893	6	30,039,432	3	*RNF39*	Body	19.38	0.000	0.350	0.508	0.158
cg12633154	6	30,039,435	3	*RNF39*	Body	18.69	0.000	0.310	0.477	0.167
cg10568066	6	30,039,442	3	*RNF39*	Body	22.29	0.000	0.372	0.560	0.188
cg16078649	6	30,039,466	3	*RNF39*	Body	13.37	0.001	0.346	0.451	0.105
cg10930308	6	30,039,476	3	*RNF39*	Body	12.69	0.001	0.193	0.317	0.125
cg01341801	6	32,489,203	4	*DRB5*	Body	4.419	0.041	0.249	0.455	0.205
cg26981746	6	32,490,012	4	*DRB5*	Body	4.645	0.036	0.501	0.633	0.132
cg12015991	6	32,490,043	4	*DRB5*	Body	5.591	0.022	0.649	0.778	0.128
cg08578320	6	32,552,039	5	*DRB1*	Body	6.558	0.014	0.754	0.588	−0.166
cg09139047	6	32,552,042	5	*DRB1*	Body	5.129	0.028	0.754	0.576	−0.179
cg15602423	6	32,552,095	5	*DRB1*	Body	8.966	0.004	0.659	0.387	−0.272
cg15982117	6	32,552,106	5	*DRB1*	Body	5.811	0.020	0.684	0.492	−0.192
cg14645244	6	32,552,205	5	*DRB1*	Body	6.068	0.017	0.521	0.318	−0.203
cg09949906	6	32,552,350	5	*DRB1*	Body	5.076	0.029	0.699	0.507	−0.192
cg10632894	6	32,552,453	5	*DRB1*	Body	4.536	0.038	0.825	0.681	−0.144
cg01053087	8	637,909	6	*ERICH1*	Body	10.55	0.002	0.330	0.164	−0.166
cg05875700	8	638,208	6	*ERICH1*	Body	8.864	0.005	0.319	0.120	−0.199
cg12641240	8	638,330	6	*ERICH1*	Body	6.055	0.018	0.194	0.069	−0.125

*DMP* differentially methylated position, *DMR* differentially methylated region

To investigate whether or not SNP genotype might be influencing methylation signal at the *RNF39* DMR, we first examined tracks on UCSC Genome Browser and determined that there were no common SNPs in the immediate vicinity of the CpGs in the DMR. Furthermore, we examined the methylation distribution for the largest effect DMP (i.e. cg10568066), which showed a relatively even spread of beta values within the MS and control groups indicating that SNP genotype is not confounding the signal (see Additional file 3).

Also within the MHC region, we identified a smaller DMR of three CpGs within a 45-bp span at the transcription start site (TSS) of the HLA complex group 4B-non-protein coding (*HCG4P6)* (Table 1). Non-MHC DMRs were also identified at *PM20D1* on Chr 1 and *ERICH1* on Chr 8. Interestingly, none of the annotated MS genes from the 110 non-HLA loci identified in the GWAS by the IMSGC [1] were represented in our extended list of 134 genes that showed CpG methylation changes as determined in this study. This may suggest that the underlying genetic and epigenetic architecture of MS is quite different outside of the MHC region.

## Discussion

In this study, we report the results of an epigenome-wide association study of methylation levels in CD4^+^ T cells of treatment-naïve female RRMS patients compared to age-matched healthy controls. In our previous study, which included patients on treatment, we found a DMR consisting of eight hypomethylated CpGs in *HLA-DRB1* [7]. In the current study, we were able to confirm five of these CpG sites, plus an additional three CpG sites clustered within the same 358-bp region. Although there is a lower effect size in the current study, the differential methylation occurs in the same direction (primarily hypomethylation). These results confirm the signal we saw in our original study and suggest this is not due to treatment effects. Another study by Bos et al. [8] did not find any changes at the *HLA-DRB1* locus. However, this study filtered all probes that had a SNP in the probe sequence on the assumption that SNPs may affect the signal at these probes. This removed all probes at *HLA-DRB1* that we find to have altered methylation status in this study. We did not remove these probes using our filters, and since the methylation signal is absent in CD8^+^ T cells derived from the same cohort, it is unlikely that SNP genotype underpins this DMR [10].

Interestingly, this study also identified an independent DMR in the MHC region in CD4^+^ T cells as being associated with MS, specifically, a large hypermethylated DMR in *RNF39*. The biological relevance of this locus can only be speculated at this stage; however, it resides within the gene body and spans an intron/exon boundary, so it is plausible that hypermethylation is involved in aberrant expression of alternately spliced transcripts or a regulatory element for nearby genes. *RNF39* is a poorly characterised gene. In rats, *RNF39* encodes a protein that plays a role in the early phase of synaptic plasticity [14].

Interestingly, one of the sites identified in the RRMS cohort (cg10568066) was also identified in a recent study that investigated the role of CpG methylation sites and ageing [15]. *RNF39* is also associated with other autoimmune conditions, such as Becet’s disease, a chronic relapsing inflammatory disease [16]. This study found a SNP near *RNF39* associated with the disease, although the functional consequences of this are not yet known. In addition, hypermethylation of 11 CpGs sites within *RNF39* was seen in naïve CD4^+^ T cells of patients with systemic lupus erythematosus (SLE) who had a history of discoid rash [17]. Furthermore, eight of these sites are the same sites as those we have identified in this study (cg10568066, cg12633154, cg13401893, cg10930308, cg03343571, cg13185413, cg09279736, cg00947782) and have similar Δbeta values to our RRMS cohort (mean_RRMS_ = 0.13; mean_SLE_ = 0.16) [17].

Although this study controlled for sex, age and treatment effects (as much as possible), there is a possibility that methylation at *RNF39* may be under the influence of other factors that were not measured in this study, e.g., exposure to Epstein-Barr virus (EBV) and smoking. A search of the literature at the time of writing did not reveal any studies reporting an association of *RNF39* methylation with known environmental risk factors of MS. However, one study did reveal an association with hepatitis B vaccination response [18] which suggests that future, larger scale studies of *RNF39* and MS should attempt to include factors such as EBV as covariates in the analysis.

## Conclusions

In conclusion, the findings from this study confirm our previous results at the MS risk locus *HLA-DRB1* and also suggest hypermethylation in an independent MHC locus, *RNF39*, is also associated with MS*.* Taken together, our results highlight the importance of epigenetic factors at the MHC locus in MS independent of treatment, age and sex. Prospective studies are now required to discern whether methylation at MHC is involved in influencing risk of disease onset or whether the disease itself has altered the methylation profile.

## Additional files

Additional file 1: DMPs and MS. List of DMPs associated with MS in CD4s. (CSV 44.8 kb)

Additional file 2: Gene set analysis of top CpG hits. Output of results from ORA analysis using Webgestalt. (XLSX 46.8 kb)

Additional file 3: Individual methylation (Beta value) distribution for top hit. Plots showing the distribution of beta values for case and control group for the top CpG in the *RNF39* gene illustrating the DMP is not due to SNP genotype. An example of genotype influenced methylation spread is also shown for comparison. (PDF 113 kb)

## Abbreviations

DMP: Differentially methylation position
DMR: Differentially methylated region
EBV: Ebstein-Barr virus
GWAS: Genome-wide association study
HC: Healthy controls
HLA: Human leukocyte antigen
MHC: Major histocompatibility complex
MS: Multiple sclerosis
PBMCs: Peripheral blood mononucleocytes
RRMS: Relapsing-remitting multiple sclerosis
SNPs: Single-nucleotide polymorphism
