# Supplementary material for: Differential methylation at MHC in CD4+ T cells is associated with multiple sclerosis independently of HLA-DRB1
Source: Clin Epigenetics. 2017 Jul 18;9:71. doi: 10.1186/s13148-017-0371-1 (PMC5516341; doi:10.1186/s13148-017-0371-1)

Individual-level beta value distribution for top hits at RNF39

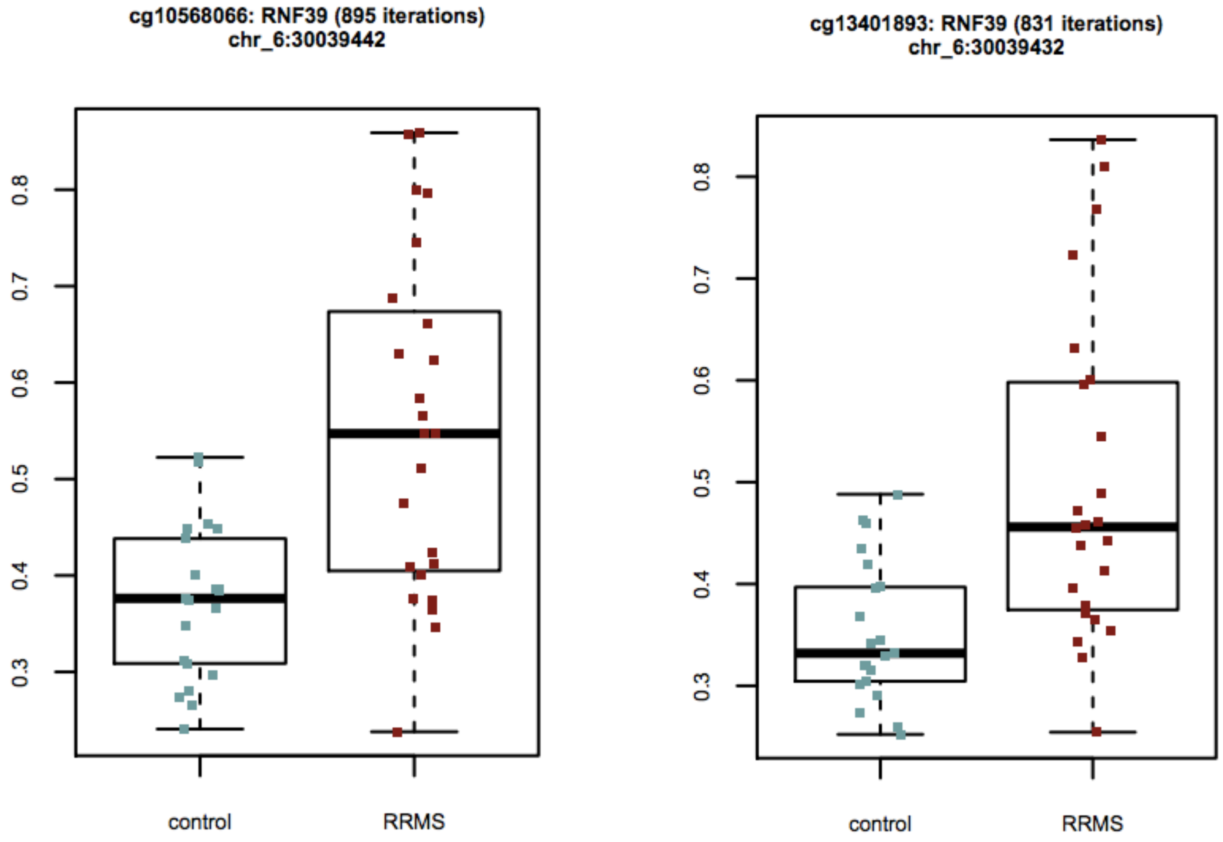

RNF39 associations <u>not due</u> to SNP genotype effect at this DMR and UCSC tracks do not show known SNP nearby

Example of SNP Effects on the distribution of CpG Methylation

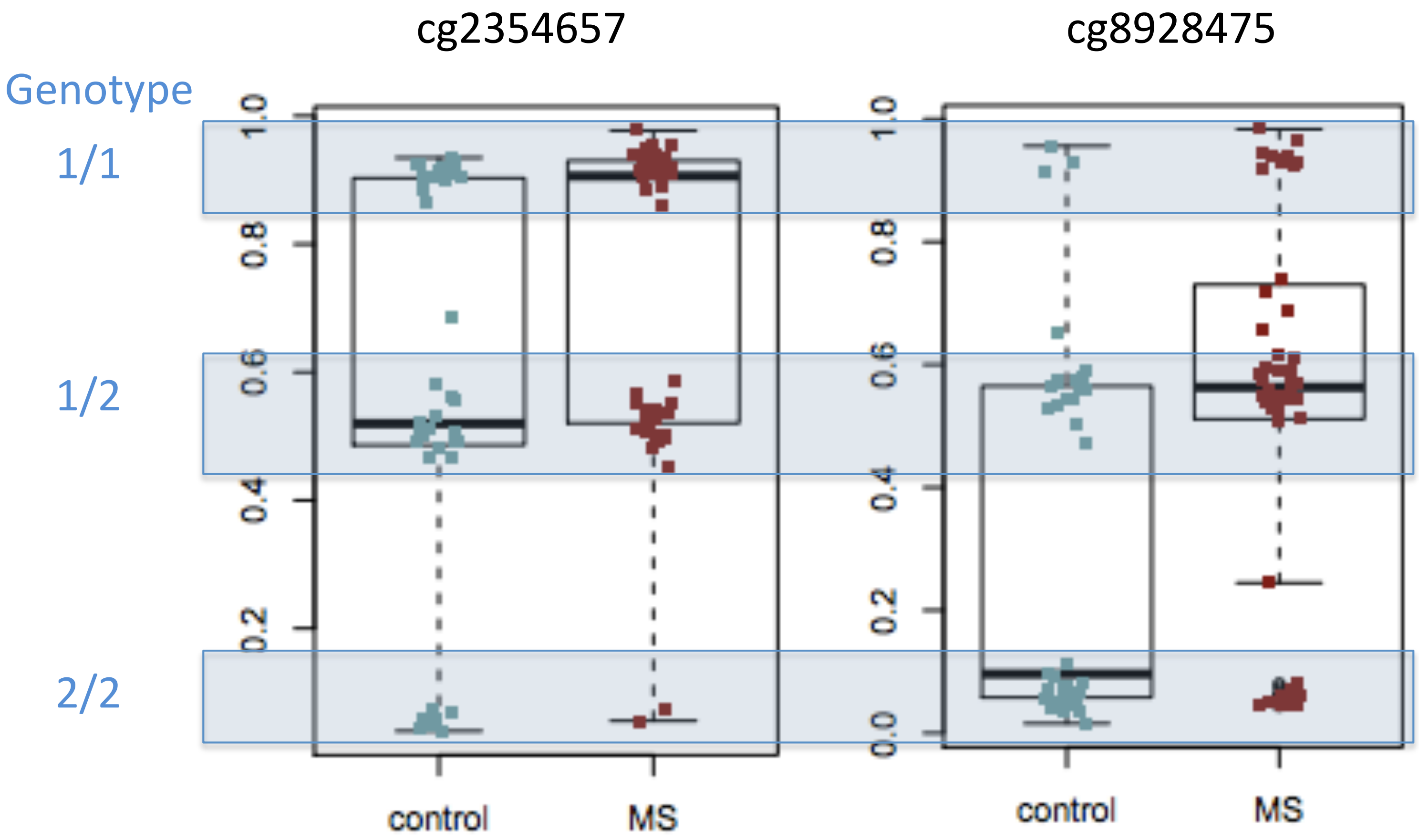

Supplement: Supplementary file 3 — Individual methylation (Beta value) distribution for top hit. Plots showing the distribution of beta values for case and control group for the top CpG in the RNF39 gene illustrating the DMP is not due to SNP genotype. An example of genotype influenced methylation spread is also shown for comparison. (PDF 113 kb) [file 13148_2017_371_MOESM3_ESM.pdf]
